# Enhanced Super4PCS Algorithm by Comparing Transformed Normals at Corresponding Points

**DOI:** 10.1155/2022/6513776

**Published:** 2022-03-30

**Authors:** Hai Liu, Shulin Wang, Donghong Zhao

**Affiliations:** ^1^Mechanical School of Jiangsu University, Zhenjiang 212013, Jiangsu Province, China; ^2^Yangzhou Polytechnic Institute, Yangzhou 225127, Jiangsu Province, China

## Abstract

In this paper, an enhanced algorithm based on the Super4PCS algorithm was established to address the problem of prolonged congruent set verification of Super4PCS for point clouds with many points or low overlap. By comparing normals of corresponding points in a source point cloud and a tentatively transformed target point cloud, this approach dramatically decreases the time required for candidate transformation verification. This strategy has been shown to improve registration efficiency in experiments.

## 1. Introduction

Point cloud registration has recently become an important method in 3D point cloud processing, which is widely utilized in virtual world reconstruction, computer-aided industrial design, medical imaging, and autonomous driving.

In practice, it is difficult to acquire a complete model directly and several views of a model should be combined, usually in the form of point clouds, to obtain a complete 3D model. This process is called registration. In detail, in the registration process, a transformation matrix is obtained to match a point cloud to another point cloud, or different views of the point cloud.

As the oldest point cloud automatic registration algorithms, the ICP algorithm and its variants [1–6] have been widely used owing to high efficiency and precision. In addition, researchers proposed point cloud registration algorithms with a probabilistic model, such as NDT [7], CPD [8], and filterReg [9], to handle outliers and noise. However, the initial position of the point clouds to be transformed is an important constraint of the algorithms, as mentioned earlier. Various coarse registration (initial alignment) algorithms were proposed to solve this problem. Fast and accurate coarse registration of point clouds is a key technology and research focus. In general, coarse registration over a point cloud is managed to find the optimal rigid transformation that can align two input point clouds to a common coordinate system. In practical applications, the data may be seriously occluded, and the overlapping area between different views of a model is minimum, making the process of finding the best rigid-body transformation challenging. Therefore, finding a fast, accurate, and robust coarse registration algorithm for point clouds is a widespread research topic.

Currently, most coarse registration algorithms for 3D point clouds can be divided into two categories or their combination: feature-based registration methods and feature-free registration methods. In the former, keypoints are extracted, and the corresponding relationship between two-point clouds is obtained using the feature descriptors that can remain unchanged under a rigid-body transformation. Next, registration is performed, for example, using the SAC-IA algorithm based on the FPFH feature [10] or the SHOT feature [11]. However, when the feature is not obvious, the noise is large, or the number of outliers is high and feature extraction is difficult to perform, limiting the registration speed and quality. Feature-free registration methods are based on exhaustive search. By traversing the entire feasible transformation space, these methods find the transformation that minimizes the error function or makes the largest number of common points shared, such as the random sample consensus (RANSAC) algorithm. These algorithms are robust, as compared with feature-based registration methods. However, these methods are unacceptably time-consuming when the number of points in the point cloud is large. This is because, when the overlapping rate is low [12], the complexity of the RANSAC algorithm is often its worst case O(*N*^3^), where *N* represents the cardinality of the target point cloud. Based on RANSAC, the four-point congruent set (4PCS) algorithm [13] and the 2PNS algorithm [14] were proposed. The method based on machine learning provides a relatively more robust transformation between two arbitrary point clouds through the invariant features generated by machine learning technology. Typical representatives of such methods include point cloud registration network (PCRNet) [15], deep global registration (DGR) [16], and a learned detector method [17]. Since learning-based methods may use more detailed features than other methods, they can be more robust to noise. However, methods based on machine learning need a lot of computing overhead in the training stage, and the interpretability of the algorithm is also poor. In addition, global registration algorithms based on heuristic algorithms belong to featureless registration methods and generally consume huge computational resources [18–21]. This paper proposes a method to improve the efficiency of Super4PCS by shortening verification time of Super4PCS comparing transformed normals.

## 2. Related Work

Aiger et al. introduced the 4PCS algorithm, which uses the geometric information of four points to compute the transformation matrix between different views. The 4PCS algorithm can run in the O(*N*^2^) time. Based on 4PCS, Mellado et al. proposed the Super4PCS algorithm, the asymptotic complexity of which is O(*N*). The key improvement in the Super4PCS algorithm is the utilization of a grid-like data structure to search for point pairs within a certain distance range.

In this paper, we improve Super4PCS by adding verifying parts of normals under a tentative transform matrix before the verification stage of original Super4PCS and eliminates fault congruent four-point sets and lowers the number of potential congruent four-point sets. In addition, we proposed a method to judge the positive and negative of normals by the convexity in the point field to ensure the positive and negative consistency of the normals at corresponding points of different views.

The time consumption of verification stage in Super4PCS accounts for a large proportion of the total time consumption. Therefore, reducing the time consumption of verification stage can obviously improve the efficiency of Super4PCS. Based on the idea, the proposed registration algorithm adds normal matching before verification and eliminates fault congruent four-point sets and lowers the number of potential congruent four-point sets. Raposo et al. presented the 2PNS algorithm [14]. The improved version of Super4PCS uses point pairs and their endpoint normal vectors instead of coplanar four points to search correspondences between views. It is experimentally showed that the 2PNS algorithm can perform registration in a shorter time than Super4PCS. However, 2PNS could fail if point clouds are too sparse or strongly dominated by sharp edges and corners, with the quality of point clouds being poor.

## 3. Review of Super4PCS

To make the description of the proposed algorithm clear, we first go over the Super4PCS algorithm briefly. The Super4PCS algorithm was established on the 4PCS algorithm, which was developed based on RANSAC. Original RANSAC would try to search exhaustively triplets because a rigid transformation may be obtained from only 3 points. Its runtime complexity is O(*N*^3^).

The 4PCS algorithm uses coplanar sets of 4 points, instead of the minimum sets of 3 points used in RANSAC, and employs a method efficiently matching affine invariant ratios in a source point cloud and a target point cloud to solve the global 3D registration problem. The 4PCS algorithm uses coplanar sets of 4 points, instead of the minimum sets of 3 points used in RANSAC, and employs a method efficiently matching affine invariant ratios in a source point cloud and a target point cloud to solve the global 3D registration problem. The algorithm selects iteratively a base set of 4 coplanar points in the source point cloud P, finds all the 4-point sets congruent with the base set in the target point cloud Q within a certain tolerance, verifies rigid transformations between the base set and its congruent 4-point sets, and retains the best transformation according to the LCP score until termination conditions are satisfied. Here, a coplanar set of 4 points consists of two two-point pairs. The asymptotic complexity of 4PCS is O(*N*^2^).

**Figure 1:**
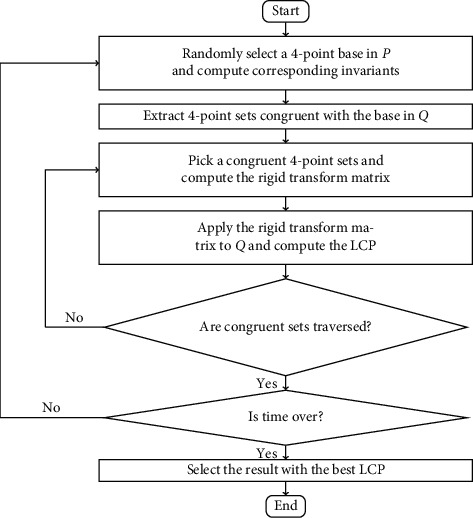
The flowchart of Super4PCS.

Super4PCS solved two main bottlenecks of 4PCS: pair extraction and elimination of nonrigid invariant 4-point candidates and decreased the asymptotic complexity to O(*N*). The target point cloud in a grid-like structure, which is subdivided recursively, is employed to compute which points are intersected with spheres centered other points in a tolerance and which point pairs are possible to make out congruent 4-point bases. The improved pair extraction is the highlight of this algorithm. In addition, Super4PCS uses hash-like structure to obtain rigid invariant 4-point candidates and avoid elimination process. The main steps of the Super4PCS algorithm are shown as Figure 1 and are as follows.

## 4. Proposed Algorithm

This section describes an improved Super4PCS algorithm which eliminates wrong transformation matrices before computing LCP scores to shorten the verification time of candidates' transformation matrices in original Super4PCS.

In detail, the proposed algorithm compares normal directions transformed using a candidate correspondence at corresponding points across different views under a candidate transformation to determine whether the correspondence is obviously fault and skip computation of LCP for fault correspondence. This screening process can avoid wasting the time of computing LCP on those obviously wrong transformation matrices and shorten the verification time of original Super4PCS. The verification phase of Super4PCS takes up a large part of the total time cost. If the time cost of this phase is reduced, the efficiency of the registration algorithm can be significantly improved.

To describe clearly, we first define a term transformed normal error here to represent the included angle between the normal of a point of a point cloud transformed by a transformation matrix and the normal of the corresponding point of the corresponding target point cloud. Using this define, the main principle of the proposed method can be described that the transformed normal error of a point in the source point cloud under a correct transformation should be very small or smaller than a certain threshold. If the transformed normal error of the transformation matrix is calculated according to a correspondence, we could exclude those transformations that cause large normal errors before the verification stage of Super4PCS. This strategy can reduce the range of potential transformations and time consumption at the verification stage of Super4PCS.

In practice, we do not need to check normal vector at all the points in the source point cloud with a transformation matrix to judge whether the transformation matrix could be optimal. The number of transformed normal errors used depends on the quality of the source point cloud and the target point cloud. If the quality of the source point cloud and the target point cloud is high, only several transformed normal errors need to be checked. The method directly using the normal error at a point in the congruent base pair of the source point cloud is an efficient way because the corresponding points of the congruent base pair of the source point cloud form the base pair of the target point cloud that does not need extra computation. If one of the several transformed normal errors under the current tentative transformation matrix is greater than a threshold, it can be considered that the current tentative transformation matrix is wrong.

**Figure 2:**
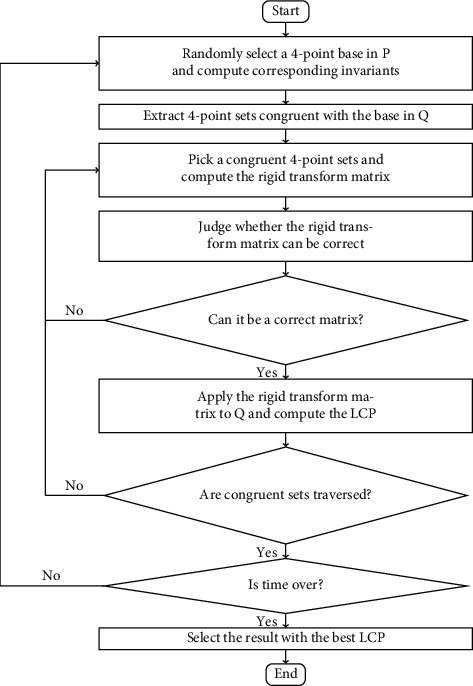
The flowchart of proposed algorithm.

**Figure 3:**
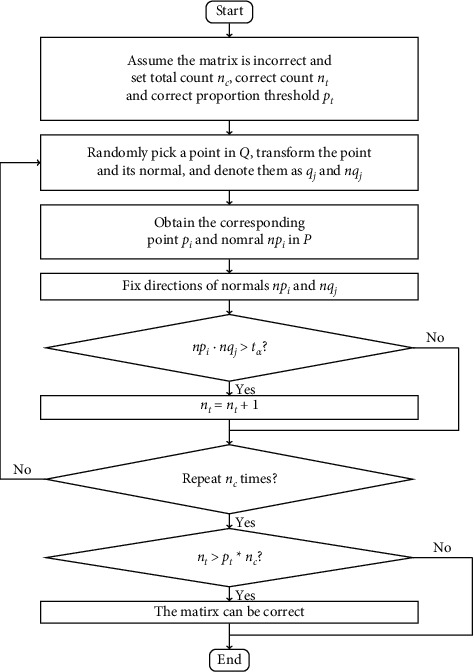
The flowchart of judging whether a transformation is fault.

For most situations, the quality of point clouds is not very high; it may not be enough to use only several transformed normal errors under a transformation. There may be a large number of points whose normal direction has great error, especially edge points, corner points, and points on scattered broken surfaces. It is unreasonable to negate a possible transformation matrix only according to the fact that the transformed normal error of one or two points exceeds the threshold. A more reasonable strategy is to check a certain number *n*_*c*_ of randomly selected transformed normal errors. The possibility that several transformed normal errors under a wrong transformation meet the requirements simultaneously is extremely small. Hence, if the proportion of points whose transformed normal errors satisfying a requirement is greater than a certain threshold *p*_*t*_, it is determined that the current tentative transformation matrix can be used in the LCP verification stage of Super4PCS. The flowchart of improved Super4PCS is showed as Figure 2. The flowchart of judging whether the point meets the requirement is shown as Figure 3.

**Figure 4:**
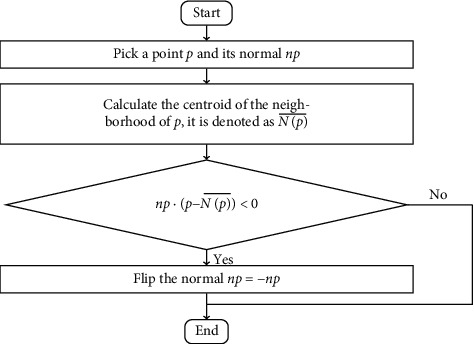
The flowchart of fixing normal directions.

Before judging whether a point meets the requirement, it is necessary to ensure the positive and negative consistency of the normals at corresponding points of different views because the calculated normal directions of the corresponding points across different views of a point cloud model may be opposite [22–24]. Here, we solve this problem according to the convexity of the neighborhood of a query point. Since the convexity of the neighborhood of a query point is consistent across different views, we can judge whether the normal direction of a point is positive or negative by calculating the cosine value of the angle between the normal and the convex direction of its neighborhood. If the cosine value is negative, we will flip the normal direction. The direction from the centroid of the neighborhood of a point to the point can be used as the convex direction of its neighborhood. The flowchart of fixing normal directions is shown as Figure 4. After fixing normal directions, whether the dot product of the normal at a point in transformed source point cloud and the normal at its corresponding point in the target point cloud is greater than a certain threshold is used to judge whether the transformed normal errors under a transformation is within the tolerance.

The total steps are shown as follows:

1. Randomly select a point pair in the target point cloud and compute corresponding invariants.
2. Extract congruent pairs with invariants of the above step in the source point cloud.
3. Select a congruent pair and compute the rigid transform matrix.
4. Randomly select *n*_*c*_ points in the source point cloud, fix their normal directions, and compute their transformed normal errors of the transformation.
5. Count the number of the points that satisfy the requirement *n*_*t*_. If the number is less than *p*_*t*_^*∗*^*n*_*c*_, jump to step 3, or go to the next step.
6. Apply the transformation and compute the LCP.
7. If all the congruent sets are traversed or the time is out, select the result with the best LCP score.

## 5. Experiments and Results

**Figure 5:**
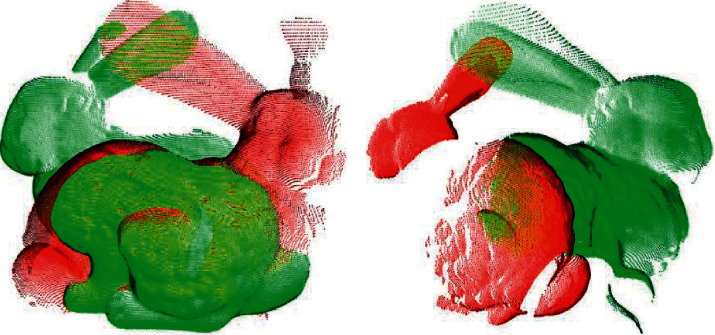
The illustration of views of the dragon model before registration. (a) Bunny 0 and bunny 45. (b) Bunny 0 and bunny 90.

**Figure 6:**
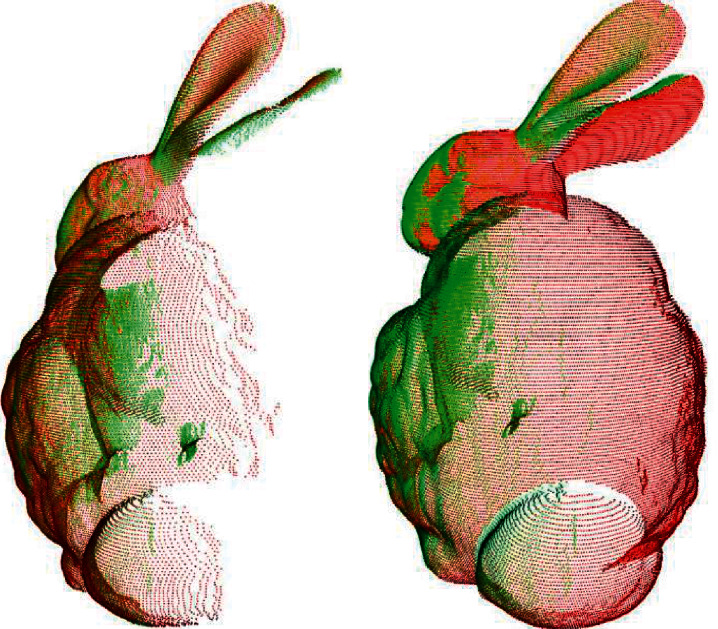
The illustration of views of the dragon model after fine registration. (a) Bunny 0 and bunny 45. (b) Bunny 0 and bunny 90.

**Figure 7:**
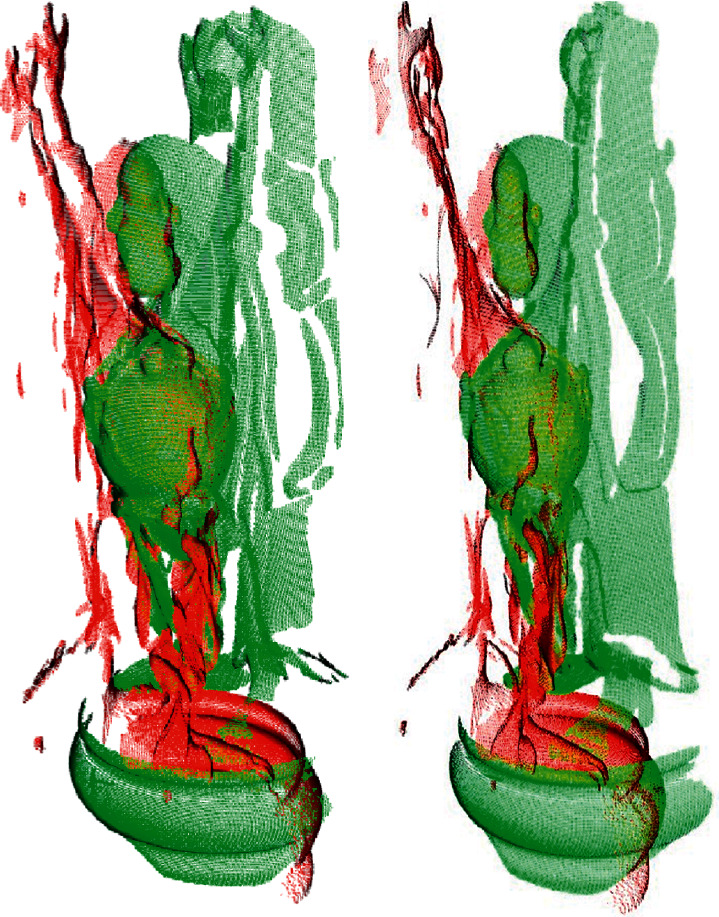
The illustration of views of the armadillo model before registration. (a) Bunny 0 and bunny 24. (b) Bunny 0 and bunny 48.

**Figure 8:**
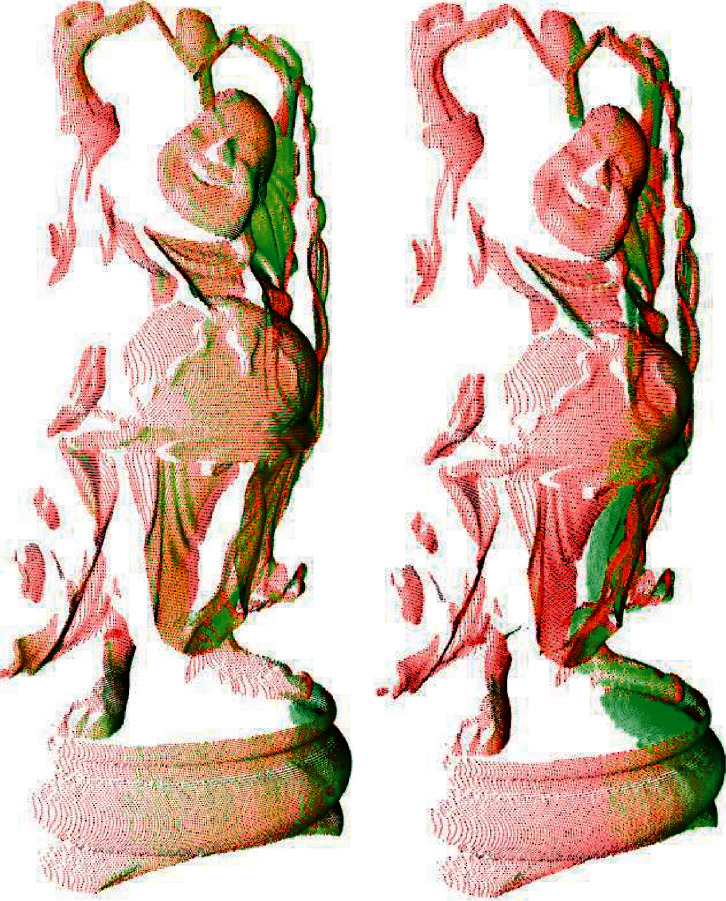
The illustration of views of the armadillo model after fine registration. (a) Bunny 0 and bunny 24. (b) Bunny 0 and bunny 48.

In this section, we performed experiments to verify the efficiency of the proposed registration algorithm. The experiments were run on different views of the bunny model, dragon model, happy buddha model, and armadillo model from the Stanford 3D Scanning Repository. These models added outliers with a variance of 0.002 mm on 5 percentage of the points. Figures 5 and 6 show the respective registration processes of the proposed algorithm over different views of the point cloud of the bunny. To show the registration processes clearly, the second views in (a) and (b) of Figure 5 are rotated by approximately 180° away from the first views.  Figure 6 shows the results obtained using the proposed algorithm. Similarly, Figures 7 and 8 show the registration process of the happy buddha model. Tables 1 and 2 show comparisons between the proposed algorithm and other algorithms on the bunny model, happy buddha model, dragon model, and armadillo model concerning time, LCP, translation error, and rotation error. The results shown in the table are the average of the results of 50 runs. The results confirm that the computation time of the proposed algorithm is significantly reduced, compared with Super4PCS and 2PNS while maintaining accuracy.

## 6. Conclusion

Registration is now commonly applied in the 3D point cloud processing field. Unlike algorithms that easily fall into the local optimum, the Super4PCS algorithm based on the 4PCS point algorithm as a global registration algorithm achieves good results. However, the time consumption of the verification of Super4PCS accounts for a high proportion of the total time. To reduce the total time of Super4PCS, an improved algorithm combining the original Super4PCS algorithm with normal matching is proposed in this paper. The method of checking the angle between normal of corresponding points in a tentatively transformed source point cloud and a target point cloud is used to obtain the candidate points of the Super4PCS algorithm to eliminate the obviously wrong transformations. The experimental results showed that this method can remarkably improve the registration efficiency.

However, the proposed algorithm is not suitable for models with a lot of broken surfaces, where a large proportion of points are difficult to estimate and obtain the correct normal direction. We plan to improve to make normal estimation robust to expand the application scope of the algorithm in the future.

## Figures and Tables

**Table 1:** The comparison between the proposed algorithm and other algorithms in time and LCP.

Method	Bunny 0° and 45°		Bunny 0° and 90°		Buddha 0° and 24°		Buddha 0° and 48°		Dragon 0° and 24°		Dragon 0° and 48°		Armadillo 0 °and 24°		Armadillo 0 °and 48°	
	Time, s	LCP, %	Time, s	LCP, %	Time, s	LCP, %	Time, s	LCP, %	Time, s	LCP, %	Time, s	LCP, %	Time, s	LCP, %	Time, s	LCP, %
Super4PCS	2.23	83.76	4.56	20.67	3.06	80.56	5.20	55.71	2.73	81.22	5.32	55.12	3.31	46.82	5.07	33.82
2PNS	1.73	85.25	3.42	20.57	1.95	80.60	4.06	57.35	2.48	81.31	4.68	55.44	2.06	47.32	4.13	34.11
Proposed algorithm	1.10	93.41	2.55	35.35	1.13	84.76	1.52	62.01	1.36	91.49	2.37	64.12	1.47	52.16	2.62	39.65

**Table 2:** The comparison between the proposed algorithm and other algorithms in translation error (TE) and rotation error (RE).

Method	Bunny 0° and 45°		Bunny 0° and 90°		Buddha 0° and 24°		Buddha 0° and 48°		Dragon 0° and 24°		Dragon 0° and 48°		Armadillo 0 °and 24°		Armadillo 0 °and 48°	
	TE, %	RE, °	TE, %	RE, °	TE, %	RE, °	TE, %	RE, °	TE, %	RE, °	TE, %	RE, °	TE, %	RE, °	TE, %	RE, °
Super4PCS	3.27	5.22	7.56	8.67	3.81	4.23	6.51	8.52	3.03	4.62	6.16	7.53	4.41	8.03	8.34	7.39
2PNS	3.51	4.83	6.32	8.37	3.35	3.77	6.24	8.12	2.85	3.78	5.63	6.87	4.05	7.47	7.41	6.78
Proposed algorithm	3.10	4.80	5.85	7.35	2.90	3.29	5.58	6.28	2.38	3.11	4.85	6.35	3.30	6.50	6.87	5.09

## Data Availability

The data used to support the findings of this study are available from the corresponding author upon request.
